# Nano selenium-doped TiO_2_ nanotube arrays on orthopedic implants for suppressing osteosarcoma growth

**DOI:** 10.3389/fbioe.2023.1252816

**Published:** 2023-09-05

**Authors:** Xiaodong Hu, Chunhai Ke, Jiaqi Zhong, Yujiong Chen, Jieyang Dong, Mingming Hao, Qi Chen, Jiahua Ni, Zhaoxiang Peng

**Affiliations:** ^1^ Affiliated Li Huili Hospital, Ningbo University, Ningbo, China; ^2^ Ningbo Institute of Innovation for Combined Medicine and Engineering (NIIME), Ningbo Medical Centre Lihuili Hospital, Ningbo University, Ningbo, China; ^3^ Ningbo Regen Biotech, Co, Ltd, Ningbo, Zhejiang, China

**Keywords:** osteosarcoma, nano selenium, TiO_2_ nanotube arrays, anti-tumor effect, orthopedic titanium implant

## Abstract

Osteosarcoma, the most common primary malignant bone tumor, is characterized by malignant cells producing osteoid or immature bone tissue. Most osteosarcoma patients require reconstructive surgery to restore the functional and structural integrity of the injured bone. Metal orthopedic implants are commonly used to restore the limb integrity in postoperative patients. However, conventional metal implants with a bioinert surface cannot inhibit the growth of any remaining cancer cells, resulting in a higher risk of cancer recurrence. Herein, we fabricate a selenium-doped TiO_2_ nanotube array (Se-doped TNA) film to modify the surface of medical pure titanium substrate, and evaluate the anti-tumor effect and biocompatibility of Se-doped TNA film. Moreover, we further explore the anti-tumor potential mechanism of Se-doped TNA film by studying the behaviors of human osteosarcoma cells *in vitro*. We provide a new pathway for achieving the anti-tumor function of orthopedic implants while keeping the biocompatibility, aiming to suppress the recurrence of osteosarcoma.

## 1 Introduction

Osteosarcoma (also called osteogenic sarcoma) is an aggressive malignant neoplasm that starts in the bones, in which the cancer cells produce malignant osteoid or immature bone tissue (Long et al., 2021). Although osteosarcoma is sensitive to chemotherapy, it frequently metastasizes to the lungs, leading to high rates of morbidity and mortality. Radical resection is still the first choice for the treatment of osteosarcoma, which requires the reconstruction of the bone defect after resection to restore the functional and structural integrity of the injured bone (Liao et al., 2021). Implantation of the metal prosthesis is one of the methods to reconstruct the bone defect site. However, even if the osteosarcoma focus is removed, the residual tumor cells could cause a high risk of recurrence of osteosarcoma after metal prosthesis implantation. Therefore, it is urgent to develop an orthopedic implant with anti-tumor function.

Recent reports reveal that anodized TiO_2_ nanotubes have a wide range of applications in surface modification of implants for improving bone integration, drug delivery, and high-throughput screening, due to their size controllability, thermal stability, corrosion resistance, and excellent biocompatibility (Brammer et al., 2010; Peng et al., 2013; Zhang et al., 2015; Chaves et al., 2016; Gulati et al., 2022; Kong et al., 2022; Sun et al., 2022). The preparation of TiO_2_ nanotube arrays on the implant surface and the loading of drugs or bioactive substances into the unique hollow structure of TiO_2_ nanotubes can promote the adhesion and growth of biological tissues in contact with the implant, and also realize the controlled release of drugs or bioactive substances, endowing the multiple functions of implants (Ayon et al., 2007; Brammer et al., 2009; Kodama et al., 2009; von Wilmowsky et al., 2009; Gultepe et al., 2010; Zhang et al., 2023). Furthermore, various surface modifications are applied to TiO_2_ nanotube arrays (TNA) to further improve the biological properties of TNA, such as inducing the generation of an apatite layer on the surface of TiO_2_ nanotubes (Oh and Jin, 2006; Kunze et al., 2008; Wang et al., 2008; Xiao et al., 2008; Kodama et al., 2009; Chen et al., 2019; Alvi et al., 2020; Wu et al., 2021), modulating the surface physicochemical properties of TiO_2_ nanotubes (Balaur et al., 2005; Lai et al., 2010; Vasilev et al., 2010), elemental doping (Yang et al., 2019; Hu et al., 2022), and alkali treatment (Oh et al., 2005). Although many studies show that appropriate surface modification of TNA can improve the biocompatibility and achieve multifunctionality, these studies mainly focus on the bone integration, and anti-tumor effect is still in its infancy, which urgently need more studies (Brammer et al., 2010; Kang et al., 2010; Lai et al., 2010; Vasilev et al., 2010; Zhao et al., 2010).

Selenium (Se), an essential trace element of the human body, is proven to be a potent anticarcinogen that aids in the prevention of bone cancer^31^, breast cancer (Harris et al., 2012), ovary cancer (Park et al., 2012), lung cancer (Tan et al., 2016), and gastrointestinal cancer (Dawsey et al., 2014). More critically, compared with conventional radiation/chemotherapy drugs with significant systematic side effects (Paus et al., 2013; Abdullah et al., 2019), selenium is demonstrated to inhibit tumor growth and protect healthy tissues (Wang et al., 2015). The anti-tumor effects of selenium are largely associated with redox metabolism, which leads to elevated intracellular reactive oxygen species (ROS) levels (Weekley et al., 2011; Misra et al., 2015). As an upstream signaling molecule, ROS plays a role in the regulation of various cellular processes, including apoptosis (Cooper, 2018; Huang et al., 2018). Some literature suggest that apoptosis shares some essential regulatory proteins in response to ROS (Cooper, 2018; Huang et al., 2018). Wang, et al. report that selenium-doped mineral nanoparticles trigger the ROS generation, causing the subsequent tumor cell apoptosis (Wang et al., 2016). However, the therapeutic window of zero-valent Se is relatively narrow, and it is difficult to precisely control its concentration, which severely limits its wide clinical application. It is reported that nano-Se is much less toxic than inorganic Se and natural organic Se, compared with general zero-valent Se (Zhang et al., 2005; Wang et al., 2007).

Based on the special structure and properties of TiO_2_ nanotube arrays, and anti-tumor effect of selenium, the researchers fabricated Se-doped TiO_2_ nanotube arrays to prepare an anti-tumor orthopedic implant (Chen et al., 2013; Cheng et al., 2017). For instance, Chen et al. fabricated Se-deposited and chitosan-coated TiO_2_ nanotubes substrates with anti-tumor, the results showed that the substrates could inhibit the proliferation of osteosarcoma cells (Chen et al., 2013). Although these strategies showed promising efficacy in inhibiting the proliferation of osteosarcoma cells, this study did not explore the effects of doped TiO_2_ nanotubes with different concentrations of Se and their surface morphology on osteosarcoma cells, as well as did not explore the mechanism of Se-doped TiO_2_ nanotubes inhibiting the proliferation of osteosarcoma cells.

Herein, We propose a novel surface modification to inhibit the growth of osteosarcoma, nano Se-doped TiO_2_ nanotube arrays were fabricated on pure titanium by anodization and electrochemical deposition. We also implement *in vitro* biological experiments to systematically investigate the influence of nano Se-doped TNA on the apoptosis of osteosarcoma cells. The results reveal that nano Se-doped TNA induce the apoptosis of osteosarcoma cells by an inherent caspase-dependent apoptotic pathway activated by the generation of reactive oxygen species (ROS). The nano Se-doped TNA in this study possesses an enhanced anti-tumor effect and excellent biocompatibility, which paves a new way for the development of orthopedic implants with anti-tumor effect.

## 2 Materials and methods

### 2.1 Material preparation and characterization

#### 2.1.1 Preparation of TNA

TiO_2_ nanotube arrays were fabricated on the surface of a pure titanium sheet by anodization. Ti sheet (5 mm × 1 mm, 99.5% purity) was used as the working electrode, while stainless steel was taken as the cathode. Before anodic oxidation, the Ti sheet was cleaned ultrasonically in acetone to remove greasy dirt. Afterwards, the Ti sheet was chemically polished for 60 s in a mixed acid solution (1:1 v/v nitric acid (HNO_3_) and hydrofluoric acid (HF)) and rinsed in deionized water. Anodic oxidation was carried out in an electrolytic cell at 20 V supplied by a regulated DC power supply (WYJ-100V5A, Stabilized Kai Power Supply, China) for 40 min. The aqueous solution containing 0.6 vol% HF was used as the electrolyte. After anodization, the surface of the Ti sheet was rinsed with deionized water and air-dried. The whole preparation process was carried out at room temperature.

#### 2.1.2 Preparation of nano Se-doped TNA

The doping of nano Se on TiO_2_ nanotube arrays was performed by electrochemical deposition. The aqueous solution containing 0.25–3 × 10^−3^ mol/L Na_2_SeO_3_ (Alfa Aster, United States) was used as the electrolyte. The pH was adjusted to about 3 by dropwise adding HF solution. The deposition voltage was 0.5 V and the deposition period was 2–10 min. After deposition, the TNA sample was rinsed with deionized water and air-dried.

#### 2.1.3 Characterization of the nano Se-doped TNA

The morphology and elemental composition of nano Se-doped TNA were characterized by field emission scanning electron microscope (FE-SEM, Hitachi SU-70, Japan), energy dispersive spectrometer (EDX, Inca Oxford, United Kingdom), X-ray photoelectron spectroscopy (XPS, Thermo Kalpha, United States), and X-ray diffraction (XRD, Philips X’Pert PRO, United States). Three groups of nano Se-doped TNA samples with Se content of 0.5wt%, 5wt%, and 22wt% (determined by EDX detection results) were UV sterilized for subsequent biological analysis.

### 2.2 Cell culture

Saos-2, L929, and MC3T3-E1 cell lineage were purchased from the National Collection of Authenticated Cell Cultures (CAS, China). Saos-2 and L929 cell lines were cultured in Dulbecco’s Modified Eagle Medium (DMEM, Corning, United States) supplemented with 10% fetal bovine serum (FBS, Gibco, United States) and 1% (v/v) anti-anti. Similarly, MC3T3-E1 was cultured in Minimum Essential Medium α (MEMα, Viva cell bioscience, China) supplemented with 10% fetal bovine serum (FBS, Gibco, United States) and 1% (v/v) anti-anti. The cells were incubated at 37°C and 5% CO_2_ in a 95% humidified cell incubator until 70%–80% confluency. Cells were dissociated with 0.25% trypsin-EDTA and seeded in microwell plates (MWP) for subsequent biological analysis.

### 2.3 Cell cytotoxicity assay

The extract of undoped TNA and 0.5 wt%, 5 wt%, and 22 wt% nano Se-doped TNA were obtained according to the international standard ISO 1099325. Briefly, samples were immersed in DMEM supplemented with 10% FBS under 37°C and 5% CO_2_ for 72 h. L929 cells (1 × 10^3^ cell/well) were seeded in 96 MWP with 150 μL of above supplemented extract and incubated for 48 h under 37°C and 5% CO_2_ atmosphere. DMEM medium with 10% FBS served as control group. 20 μL of 5 mg/mL of 3-(4,5-dimethylthiazol-2)-2,5-diphenyltetrazolium bromide (MTT, Sigma, United States) was added into each well, followed by incubation at 37°C for 3 h to determine succinate dehydrogenase activity.

After the mixture of medium and MTT were removed, the methanogenic product was solubilized in 100 µL of isopropanol solution containing 0.04 mol/L HCl. Absorption at 570 nm was measured using an automated plate reader (Perkin-Elmer) for quantitative detection.

### 2.4 Hemolytic tests

Undoped TNA and nano Se-doped TNA (0.5 wt%, 5 wt%, 22 wt%) served as test groups, and Triton X-100 (1%) and 0.9% normal saline (NS) were employed as positive control group (PC) and negative control group (NC), respectively. Each sample was put into a centrifuge tube, and then red blood cell (RBC) saline resuspension was added into each tube. All specimens were incubated at 37°C for 3 h and then were centrifuged at 1,600 rpm for 8 min to obtain the supernatant. The absorbance of the supernatant was measured at 545 nm. The hemolysis rate was calculated according to formula (1).

$$Hemolysis\;rate\;\left(\%\right)=\frac{\left(A_{s}-A_{nc}\right)}{\left(A_{pc}-A_{nc}\right)}\times 100\%$$ (1)

Where A_s_, A_nc_, and A_pc_ were the absorbance values of the experimental group, negative control group, and positive control group, respectively.

### 2.5 Cell proliferation test

The effect of nano Se-doped TNA with different Se concentrations (0.5wt%, 5wt%, 22wt%) on the proliferation of Saos-2 cells was tested using the AlamarBlue reagent (Thermofisher, United States) according to the manufacturer’s instructions. Briefly, undoped TNA and 0.5 wt%, 5 wt%, and 22 wt% nano Se-doped TNA were placed in a 96 MWP. 20 μL of AlamarBlue was added into 1 × 10^5^ cells/mL Saos-2 cell suspension to mix well, and then 180 μL cell suspension was added into each well, followed by incubation for 24 h at 37°C and 5% CO_2_ atmosphere. The reduction of AlamarBlue was measured by microspectrophotometer and the reduction rate of AlamarBlue was calculated using the formula (2) provided by the manufacturer’s protocol.

$$Reduced\;\left(\%\right)=\frac{{\left(\varepsilon_{ox}\right)}_{\lambda_{2}}\cdot A_{\lambda 1}-{\left(\varepsilon_{ox}\right)}_{\lambda_{1}}\cdot A_{\lambda 2}}{{\left(\varepsilon_{Red}\right)}_{\lambda 1}\cdot {A’}_{\lambda 2}-{\left(\varepsilon_{Red}\right)}_{\lambda 2}\cdot {A’}_{\lambda 1}}\times 100\%$$ (2)

Where λ_1_ = 570 nm, λ_2_ = 600 nm; ε_ox_ was the molar extinction coefficient of the oxidized form of AlamarBlue (blue), ε_ox_ 570 = 80586, ε_ox_ 600 = 117216; ε_red_ is the molar extinction coefficient of the reduced form of AlamarBlue (pink), ε_Red 570nm_ = 155677, ε_Red 600nm_ = 14652. A indicates the absorbance value, and A′ denotes the absorbance value of the negative control.

We also performed Cell Counting Kit-8 (CCK-8, Bimake, China) assay to investigate the effect of nano Se-doped TNA with different Se concentrations (undoped, 0.5 wt%, 5 wt%, 22 wt%) on the proliferation of MC3T3-E1 cells. MC3T3-E1 cells (2 × 10^3^ cell/well) were cultured on the surface of undoped, 0.5 wt%, 5 wt%, 22 wt% nano Se-doped TNA and blank well (control group) for 24 h.

### 2.6 Cell adhesion

MC3T3-E1 (3 × 10^3^cells/well) and Saos-2 (3 × 10^3^cells/well) were cultured on undoped TNA and 0.5 wt%, 5 wt%, and 22 wt% nano Se-doped TNA for 24 h. MC3T3-E1 and Saos-2 cells cultured on the samples were fixed with 2.5% glutaraldehyde and permeabilized with 1% Triton X-100. F-actin and nuclei were stained with Fluorescein isothiocyanate (FITC)-phalloidin and DAPI (Solarbio, Beijing, China), respectively, according to the manufacturer’s protocol. Cellular morphology was observed by confocal laser scanning microscopy (CLSM, LEICA TCS SP8, Germany).

### 2.7 Cell apoptosis

The exact percentage of the apoptotic Saos-2 cells was analyzed with Annexin V- FITC/PI staining. Saos-2 cells were cultured onto the surface of undoped TNA and 0.5 wt%, 5 wt%, and 22 wt% nano Se-doped TNA (3 × 10^5^/well) for 24 h, and then the Saos-2 cells were collected and stained with Annexin V- FITC/PI (Solarbio, Beijing, China). Flow cytometer (FCM, Beckman Coulter, United States) was used to determine the fluorescence signal of Saos-2 cells.

Saos-2 cells (10^4^cells/well) were seeded on the surface of undoped TNA, 0.5 wt%, 5 wt%, and 22 wt% nano Se-doped TNA, incubated for 24 h, and then stained with Hoechst 33258 (Solarbio, China) and acridine orange/ethidium bromide (AO/EB) according to manufacturer’s protocols, respectively. Apoptotic cells were examined using a fluorescent microscope (Olympus, Tokyo, Japan) within 30 min.

### 2.8 Detection of reactive oxygen species (ROS)

ROS Assay Kit (Beyotime, Shanghai, China) was used to detect the levels of ROS production following the manufacturer’s protocol. Briefly, Saos-2 cells cultured on the surface of TNA groups for 24 h were collected by trypsin digestion and stained by 2,7-Dichlorodihydrofluorescein diacetate (DCFH-DA) ROS sensitive probe. The intracellular ROS content was evaluated using flow cytometer (FCM, Beckman Coulter, United States) and confocal laser scanning microscopy (CLSM, LEICA TCS SP8, Germany).

### 2.9 qPCR analysis

Saos-2 cells (4 × 10^5^/well) were cultured onto the surface of undoped TNA and 0.5 wt%, 5 wt%, and 22 wt% nano Se-doped TNA for 24 h. The total RNA of saos-2 cells was extracted using TRIzol Reagent (Thermofisher, United States), and the complementary DNA (cDNA) was synthesized using the total RNA with the TransScript^®^ All-in-One First-Strand cDNA Synthesis SuperMix for qPCR (TransGen, China) according to manufacturer instructions. The expression levels of the apoptosis-related genes (*BAX*, *CYTC*, *CASP9*, *CASP8*, *CASP3*) were detected by reverse transcription-polymerase chain reaction (RT-PCR) assay, and the primers’ sequences were shown in Table 1. *GAPDH* was chosen as the reference gene in this study.

**TABLE 1:** Primers’ sequences used in real-time PCR.

Target gene	Sequences	
	Forward primer (5′→3′)	Reverse primer (5′→3′)
*BAX*	CCC​GAG​AGG​TCT​TTT​TCC​GAG	CCA​GCC​CAT​GAT​GGT​TCT​GAT
*CYTC*	CTT​TGG​GCG​GAA​GAC​AGG​TC	TTA​TTG​GCG​GCT​GTG​TAA​GAG
*CASP8*	AGA​GTG​AGG​CGA​TTT​GAC​CTG	GTC​CGA​AAC​AAG​GTG​AGG​GTT
*CASP9*	CTC​AGA​CCA​GAG​ATT​CGC​AAA​C	GCA​TTT​CCC​CTC​AAA​CTC​TCA​A
*CASP3*	CAT​GGA​AGC​GAA​TCA​ATG​GAC​T	CTG​TAC​CAG​ACC​GAG​ATG​TCA
*GAPDH*	ACC​CAC​TCC​TCC​ACC​TTT​GAC	TGT​TGC​TGT​AGC​CAA​ATT​CGT​T

### 2.10 Statistical analysis

All statistical analyses were performed using SPSS 20.0 (SPSS, Chicago, IL, United States). Data was expressed as mean ± standard deviation (SD) (*n* = 3). Differences among groups were analyzed and determined by Student’s *t*-test or one-way ANOVA followed by Tukey’s *post hoc* test or Bonferroni *post hoc* test. The *p*-value less than 0.05 was considered statistically significant.

## 3 Result and discussion

### 3.1 Fabrication and characterization of nano Se-doped TNA

**FIGURE 1:**
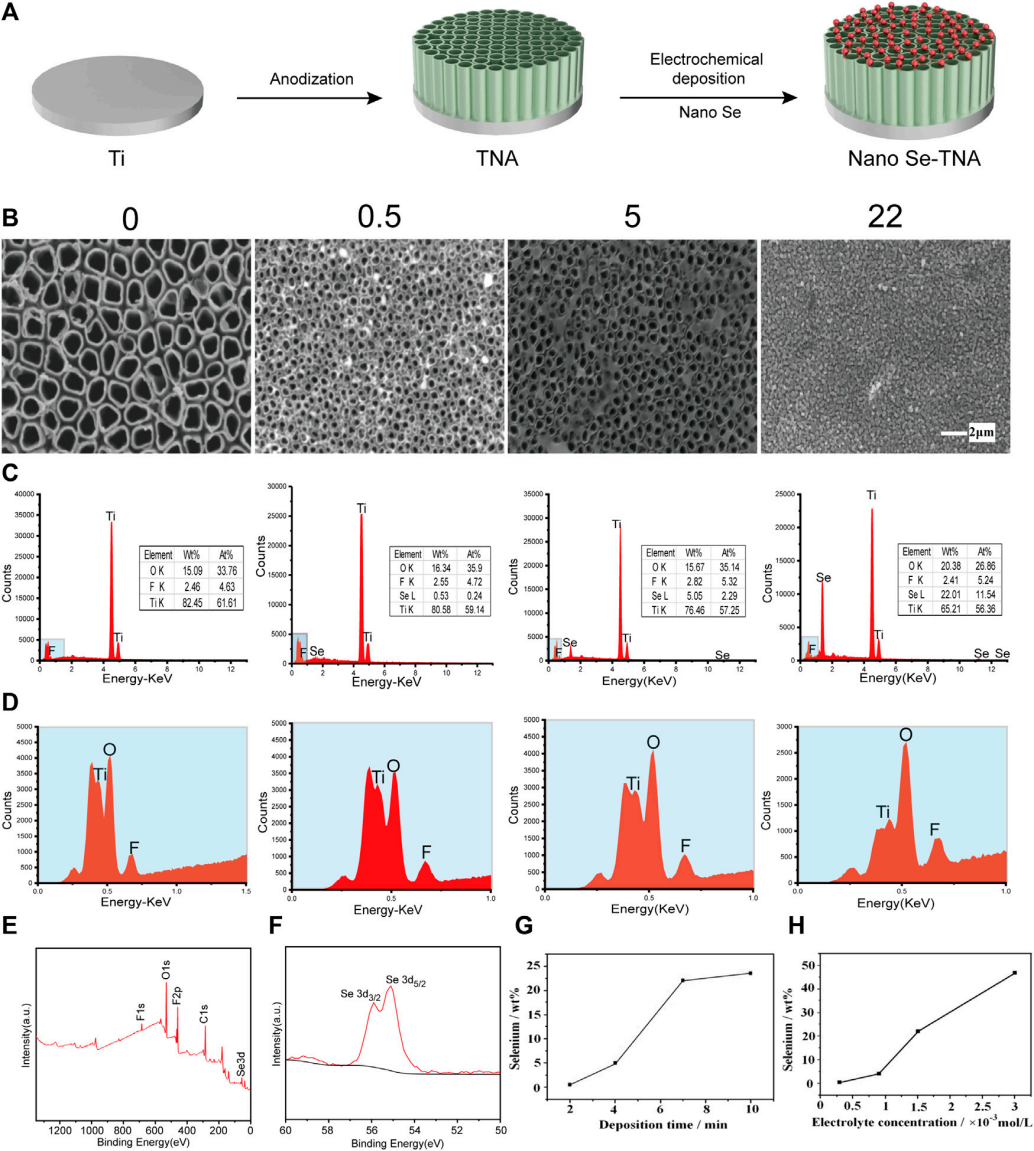
Surface characteristics of nano Se-doped TNA. **(A)** Schematic diagram of preparation process of nano Se-doped TiO_2_ nanotube arrays; **(B)** Surface morphology of undoped TNA and 0.5 wt%, 5 wt%, and 22 wt% nano Se-doped TNA, scale bar = 2 μm; **(C,D)** Element composition and content of undoped TNA and 0.5 wt%, 5 wt%, and 22 wt% nano Se-doped TNA; **(E)** XPS spectrum of 5wt% nano Se-doped TNA; **(F)** Se 3d high-resolution spectra of 5wt% nano Se-doped TNA detected by XPS; **(G)** Relationship between the deposition time and selenium content; **(H)** Relationship between the electrolyte concentration and selenium content.

Figure 1A shows the preparation process of nano Se-doped TiO_2_ nanotube arrays: TiO_2_ nanotube arrays are fabricated on the surface of pure titanium by anodization, and then, nano Se is deposited on TiO_2_ nanotube arrays by electrochemical deposition. The surface morphology and composition of nanostructured materials is crucial to the actual function of the material. The surface morphology of undoped TNA and nano Se-doped TNA are shown in Figure 1B. The undoped TNA are highly ordered and uniform in size, and the diameter of nanotubes is about 120 nm and the length of nanotubes is about 400 nm. The surface of 0.5wt% nano Se-doped TNA with 2 min deposition is covered by some nano scaled fine deposits with sizes around 100 nm, and the structure of TNA remained intact. It can be observed that the surface of 5wt% nano Se-doped TNA with 4 min deposition is locally covered by nanoflakes of irregular shape and size about 100 nm, which evenly and individually disperse on the surface of the TNA, and the flakes are confirmed to be Se elements by EDX test. There are still some uncovered parts of TiO_2_ nanotube array, and the morphological structure remained clear and consistent. When the electrochemical deposition time was extended to 7 min, a layer of reddish-brown deposit on the surface of the sample could be observed by naked eye, and the surface of 22 wt% nano Se-doped TNA was completely covered and the nanotubes were not visible. The deposited Se agglomerated into 300–400 nm lumpy particles and there were very obvious cracks among the lumpy particles. When the deposition time was 10 min, the surface morphology of the TiO_2_ nanotube array was similar to 22 wt% nano Se-doped TNA.

Lowering the pH value of the electrolyte enables the hydrolysis reaction to proceed more rapidly and adequately. Moreover, we believe that the special porous structure of the TiO_2_ nanotube array also facilitates the deposition of selenium. The porous structure of TiO_2_ nanotube array provides a fixation point for the deposition and merger of Se. At the initial stage of electrochemical deposition, nano Se particles are attached and immobilised at certain points on the surface of the TiO_2_ nanotube arrays, and these nanoscale particles gradually agglomerate to form blocks and sheets.

The EDX spectra of the surface of nano Se-doped TNA are shown in Figures 1C, D. In addition to the peaks of Ti, O, and F elements, there is Se peak in the spectrum, which proves that Se has been successfully deposited on the TiO_2_ nanotube arrays. The content of various elements of nano Se-doped TNA fabricated in this work is shown in Table 2. Nano Se-doped TNA mainly contains Ti, O and Se elements. The selenium content is 0.53 ± 0.03wt% (0.5 wt% nano Se-doped TNA), 5.17 ± 0.11 wt% (5wt% nano Se-doped TNA), and 22.19 ± 0.19wt% (22 wt% nano Se-doped TNA), respectively. The XPS spectrum of 5 wt% nano Se-doped TNA are presented in Figure 1E. XPS spectra in Figure 1F show two peaks at 55.21 eV and 55.9 eV corresponding to Se 3d_5/2_ and Se 3d_3/2_, the binding energies are similar to the 3d_5/2_ and Se 3d_3/2_ peaks of monatomic Se at 55.2 eV and 55.9 eV, respectively, and the results suggest that Se is deposited as a monomer in the whole row of TiO_2_ nanotube arrays (Shalvoy et al., 1977; Shenasa et al., 1986). Supplementary Figure S1 shows a typical X-ray diffraction pattern of 5 wt% nano Se doped TNA, which reveals that the polymorph of the obtained nano Se is amorphous Se (Li et al., 2010). The concentration and morphology of the deposited Se element on the TiO_2_ nanotube arrays can be adjusted by changing the electrochemical deposition time and the concentration of the electrolyte. Figure 1G depicts the trend of Se concentration deposited on the TiO_2_ nanotube arrays with increasing deposition time when the electrolyte concentration is fixed (taking 1.5 × 10^−3^ mol/L Na_2_SeO_3_, for example). It is clear that the Se concentrations are positively associated with increasing deposition time: Se content is around 0.5wt% when the deposition time is 2 min, and Se content is around 5 wt% when the deposition time is 4 min, and around 22 wt% of Se when the deposition time is 7 min, and around 24 wt% of Se when the deposition time is 10 min. It can also be seen from Figure 1G that the deposition rate of Se on the nanotubes at the beginning stage is higher than that at the later stage. Taking the results (Figures 1C, G) into consideration, we speculate that nano Se is scattered on the surface of the nanotubes at the beginning, and then the dispersed nano Se grow up gradually and interconnect when the deposition time exceeds a certain value. Eventually, Se agglomerates to cover the whole surface of TNA, and the surface of TNA shows obvious reddish-brown color. As the deposition time continues to extend, the reddish-brown particles floating in the electrolyte around the sample could be observed by nude eye, indicating the deposition of Se on the surface of nanotubes has reached saturation state. Figure 1H shows the trend of the deposition concentration of Se on the TiO_2_ nanotube arrays with the electrolyte concentration when the deposition time is fixed at 7 min. The concentration of Se is around 1wt% when the electrolyte concentration is 0.25 × 10^−3^ mol/L, and 5wt% of Se when the electrolyte concentration is 0.9 × 10^−3^ mol/L, and 22wt% of Se when the electrolyte concentration is 1.5 × 10^−3^ mol/L, and Se concentration is as high as 47wt% when the electrolyte concentration is 3 × 10^−3^ mol/L, which indicates that the concentration of Se deposited on the surface of TNA increases as the electrolyte concentration increases. The deposition rate of Se in the high-concentration electrolyte is much higher than that in the low-concentration electrolyte. The floating reddish-brown particles are visible in the electrolyte around the sample, implying Se deposition on the surface of TNA reaches saturation.

**TABLE 2:** The element composition and concentration of Se-doped TNA.

Samples	Ti	O	Se
0.5 wt% nano Se-doped TNA	80.08 ± 1.08 wt%	16.70 ± 0.75 wt%	0.53 ± 0.03 wt%
5 wt% nano Se-doped TNA	78.12 ± 2.72 wt%	14.85 ± 1.19 wt%	5.17 ± 0.11 wt%
22 wt% nano Se-doped TNA	66.70 ± 1.84 wt%	9.30 ± 1.49 wt%	22.19 ± 0.19 wt%

### 3.2 *In vitro* biocompatibility evaluation of nano Se-doped TNA

**FIGURE 2:**
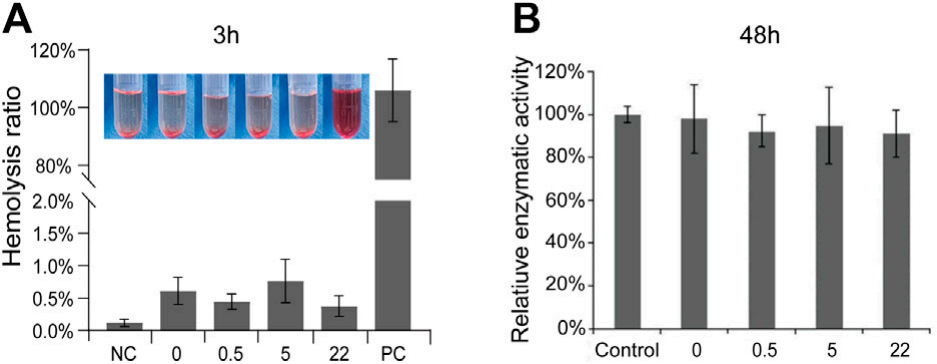
Biocompatibility assessment. **(A)** Hemolysis rates and correlation images of TiO_2_ nanotube arrays doped with different Se contents. **(B)** Relative enzymatic activity of L-929 cells in cytotoxicity testing. 0, 0.5, 5, 22 represent undoped TNA, 0.5 wt% nano Se-doped TNA, 5wt% nano Se-doped TNA, 22 wt% nano Se-doped TNA, respectively. *n* = 5 independent samples, *p < 0.05 by one-way ANOVA.

A novel biomedical material must possess good biocompatibility (Shirzaei Sani et al., 2019). As an potential orthopedic implant material, the nano Se-doped TNA will inevitably contact with blood. Therefore, we conduct hemolysis tests to assess the hemocompatibility of nano Se-doped TNA. The standard specifies the materials with hemolysis rate (HR) of 2%–5% and >5% as slightly hemolytic and hemolytic, respectively, whereas the materials with 0%–2% of hemolysis are categorized as non-hemolytic (Leitão et al., 2013). As shown in Figure 2A, the bright red represent the positive control group (PC), whereas all TNA groups appear colorless, similar to the negative control group (NC). The hemolysis rates of all TNA groups are less than 2%, indicating that nano Se-doped TNA has excellent hemocompatibility. Moreover, the cytocompatibility of the nano Se-doped TNA is evaluated by MTT testing the enzymatic activity of mouse-derived fibroblasts (L929) cultivated into the extracts of all TNA samples for 48 h. There is no significant difference in the enzymatic activity among all TNA groups and the control group, indicating that the extracts of 0.5wt% Se, 5wt% Se, and 22wt% Se nano Se-doped TNA have no cytotoxic effect (Figure 2B). Overall, the increased content of deposited nano Se does not affect the hemocompatibility and cytocompatibility of TNA.

### 3.3 Effect of nano Se-doped TNA on the cell proliferation

**FIGURE 3:**
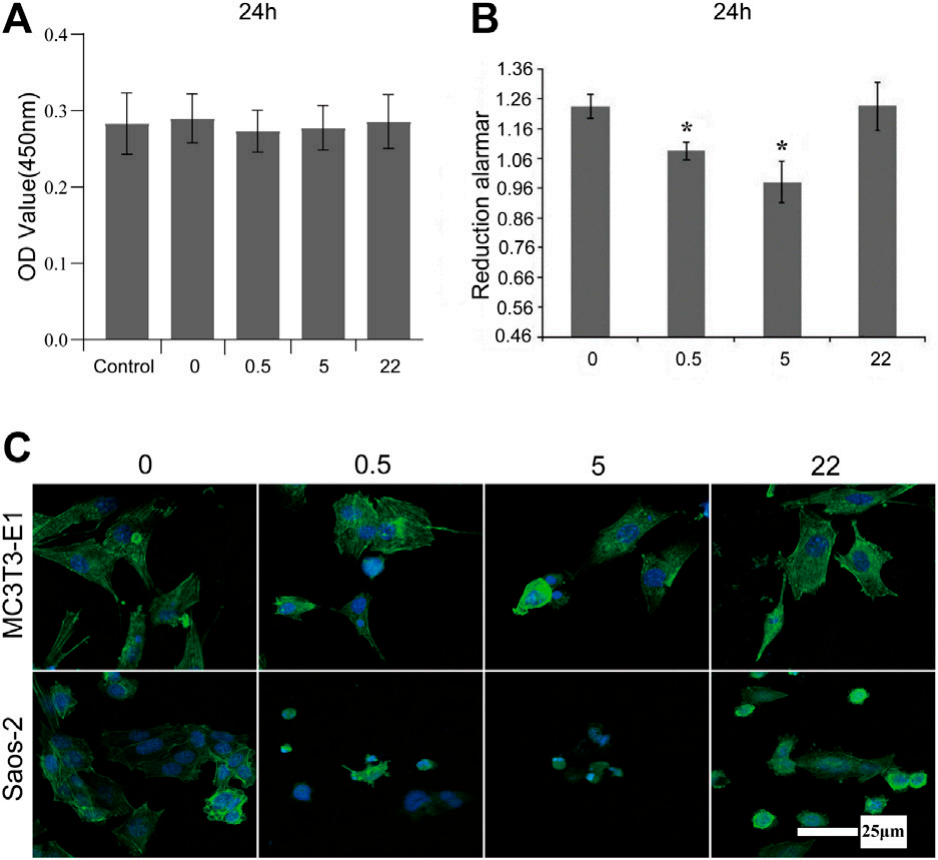
Proliferation and morphology of MC3T3-E1 and Saos-2 cell on the surface of nano Se-doped TNA. **(A)** CCK-8 assay for the proliferation of MC3T3-E1 cultured on the surface of TNA samples for 24 h; **(B)** Reduced alamar values of osteosarcoma Saos-2 cells cultured on the surface of TNA samples for 24 h; **(C)** Confocal laser scanning microscopy (CLSM) images of MC3T3-E1 and Saos-2 cells stained with FITC-phalloidin (green) and DAPI (blue) after culturing on the surface of TNA samples for 24 h. Scale bar = 25 μm. (0, 0.5, 5, 22 represent undoped TNA, 0.5 wt% nano Se-doped TNA, 5wt% nano Se-doped TNA, 22 wt% nano Se-doped TNA, respectively). *n* = 5 independent samples, *p < 0.05 by one-way ANOVA.

The mouse preosteoblast cell line (MC3T3-E1) and the human osteosarcoma cell line (Saos-2) are cultured on the surface of undoped TNA and nano Se-doped TNA to investigate the effect of nano Se-doped TNA on the proliferation of MC3T3-E1 and Saos-2. Figure 3A shows the proliferation of MC3T3-E1 cultured on the surface of nano Se-doped TNA (test groups) and DMEM cell culture media (control group). There is no any significant difference in the proliferation of MC3T3-E1 cells between all groups, which is consistent with the results of Figure 2B. As seen in Figure 3B, the proliferation of Saos-2 cell seeded on 0.5wt% and 5wt% nano Se-doped TNA is significantly lower than that of the undoped TNA and 22wt% Se-doped TNA. Among them, Saos-2 cells on the 5 wt% nano Se-doped TNA show the lowest active proliferation. Furthermore, fluorescence staining is carried out to observe the cell morphology cultured for 24 h on the surface of undoped TNA and nano Se-doped TNA (Figure 3C). MC3T3-E1 cells on all groups show very high cell confluence and intact morphology. However, Saos-2 cells on 0.5 wt% and 5 wt% nano Se-doped TiO_2_ TNA exhibit extremely low cell confluence and cytoskeletal abnormalities, compared with undoped TNA and 22 wt% Se-doped TNA, demonstrating that 0.5 wt% and 5 wt% nano Se-doped TiO_2_ TNA significantly inhibit the proliferation of Saos-2 cell lineage.

The surface morphology also alters the celluar response. For MC3T3-E1 cells, the unique structure of TiO_2_ nanotubes facilitates proliferation, adhesion and differentiation of preosteoblasts (Brammer et al., 2012). Therefore, MC3T3-E1 adhered easily on the surface of the nanotube incompletely covered group (0.5 wt% and 5 wt% nano Se-TNA), while 22 wt% nano Se-doped TNA was a rough surface, and previous studies have shown that roughness is favourable for cell proliferation and adhesion (Faia-Torres et al., 2014; Han et al., 2020). Compared with the Control group, the number of cells, cellular integrity, and adhesion in the Se-doped TNA group did not differ significantly. In addition, nano-selenium had no significant effect on MC3T3-E1 proliferation, and adhesion. There was no significant difference in cell number, cell integrity and adhesion in the Se-doped TNA group compared with the Control group, nano Se had no significant inhibitory effect on MC3T3-E1 (Cheng et al., 2017). For Saos-2 cells, compared with the uniform surface of TiO_2_ nanotube array, the 0.5wt and 5wt% nano Se-doped TNA surfaces were covered with un-interconnected tiny flakes of nano selenium, and osteosarcoma cells planted on the 0.5wt and 5wt% nano Se-doped TNA surfaces would be up taken by the cell in the cytosolic form of nano selenium and eventually induced a significant inhibitory effect on Saos-2 cells (Dai et al., 2021). Around the organelles to play a role, and eventually induced apoptosis in Saos-2 cells. On the other hand, the surface of 22 wt% nano Se-doped TNA was covered with interconnected block particles of nano selenium, which prevented the tumor cells from uptaking the block particles of nano selenium, so the inhibition of tumor cells by nano Se at this concentration was relatively small (Zhang et al., 2009).

### 3.4 Apoptosis in osteosarcoma cells induced by nano Se-doped TNA

**FIGURE 4:**
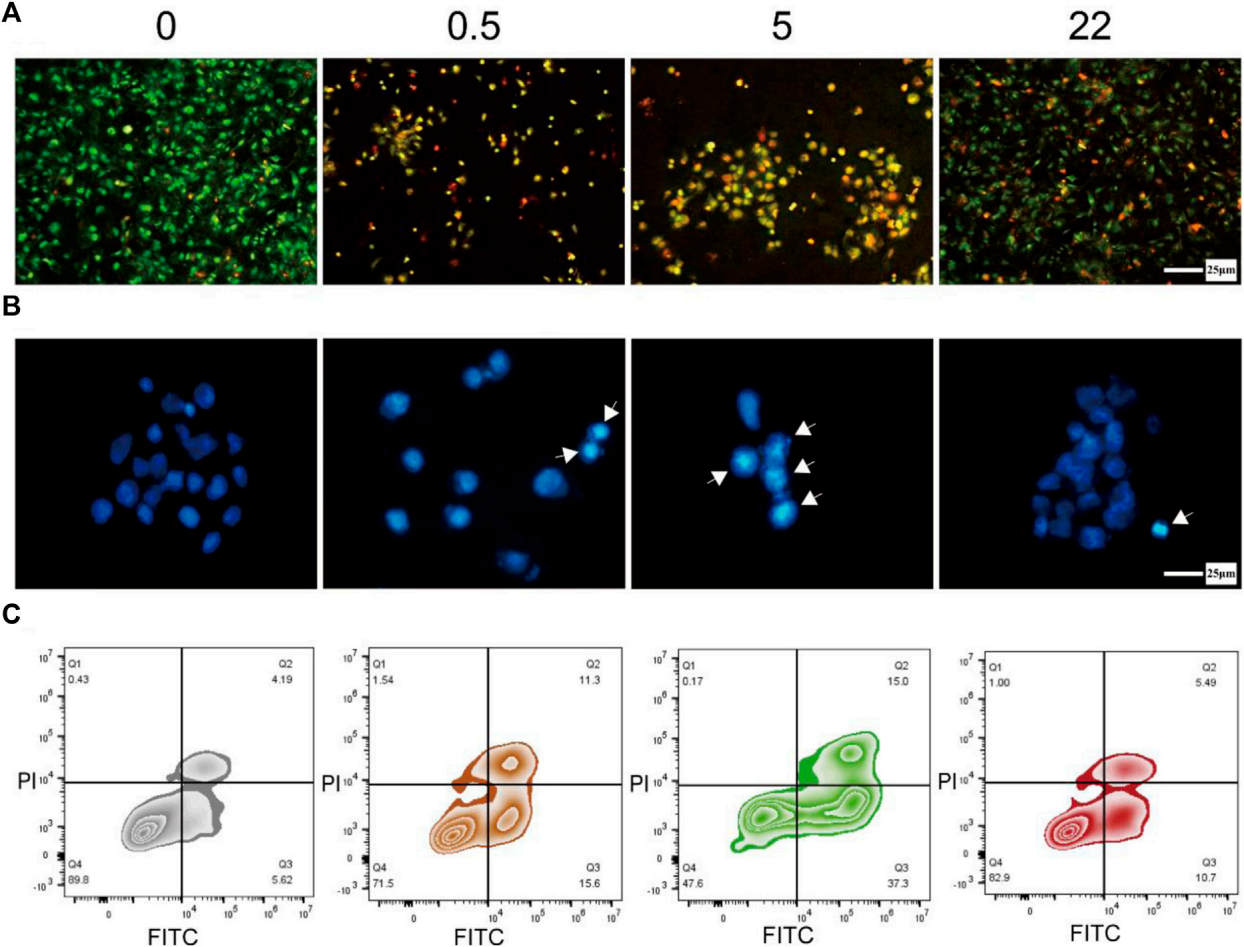
Induction of apoptosis in osteosarcoma cells. **(A)** Fluorescence microscopy image of Saos-2 cells doubly stained with AO/EB after culturing on the surface of TNA samples for 24 h (green: living cell, yellow: apoptosis cell, red: dead cell). Scale bar = 25 μm. **(B)** Fluorescence microscopy images of Saos-2 cells stained with Hoechst 33258 after culturing on the surface of TNA samples for 24 h (The cell nucleus in apoptotic are labeled by white arrows). Scale bar = 25 μm. **(C)** Annexin V-FITC/PI double staining of Saos-2 cells incubated on the surface of TNA samples for 24 h. (0, 0.5, 5, 22 represent undoped TNA, 0.5 wt% nano Se-doped TNA, 5 wt% nano Se-doped TNA, 22 wt% nano Se-doped TNA, respectively). *n* = 3 independent samples, *p < 0.05, **p < 0.01, compared with undoped TNA group by one-way ANOVA.

Previous studies have shown that the mechanism of Se inhibiting tumor cell viability is mainly inducing the apoptosis of tumor cells (Wang et al., 2016; Li et al., 2020), particularly those mediated by ROS (Chen et al., 2012). Here, we hypothesize that nano Se**-**doped TNA may effectively inhibit tumor cells proliferation by promoting cancer cell apoptosis. Therefore, we used Hoechst 33258 staining, AO/EB double staining, and Annexin V-FITC/PI double staining flow cytometry to verify our hypothesis. The AO/EB double-stained fluorescence microscopy images (Figure 4A) show the effect of the nano Se-doped TiO_2_ nanotube arrays on the viability and apoptosis of Saos-2 cells. The living cells cover the whole surface of undoped TNA group in Figure 4A, indicating that Saos-2 cells have high viability on the surface of the undoped TNA. The apoptotic and dead cells almost occupy the whole surface of 0.5wt% and 5wt% Se-doped TNA groups and the living cells are almost undetectable, indicating that the Saos-2 cells on these two groups are hard to getting survive, and these two groups significantly reduce the survival rate of Saos-2 cells (Figure 4A). The live, apoptotic and dead Saos-2 cells coexist on the surface of 22 wt% Se-doped TNA group, but the living cells are still in the majority, indicating that the survival rate of Saos-2 cells on the 22 wt% Se-doped TNA group is higher than that on the 0.5 wt% and 5 wt% Se-doped TNA groups, but still lower than that on the undoped TNA group. It can be concluded that the nano Se deposited on the surface of TNA possesses a strong inhibitory effect on the viability of Saos-2 cells, and however, higher Se doping contents do not exert a stronger inhibitory effect on the viability of Saos-2 cells. Only an appropriate concentration of doped nano selenium has a significant inhibitory effect. The results of Figure 4A are consistent with those shown in Figures 3B, C.

We further investigate the nuclear morphology of Saos-2 cells stained with Hoechst 33258 by fluorescence microscopy (Figure 4B). The nuclei of Saos-2 cells on all nano Se-doped TNA groups exhibit brighter blue staining and more concentrated morphology compared to that on the undoped TNA, indicating the deposited nano Se enhances the cell apoptosis. The cell nuclei on the surface of 0.5 wt% and 5 wt% nano Se-doped TNA groups show brighter blue staining and more concentrated morphology than that on 22 wt% Se-doped TNA, implying that an appropriate concentration of doped nano Se significantly promotes cell apoptosis.

Next, we implement Annexin V-FITC/PI double-staining assay in order to quantify the apoptosis rate of Saos-2 cells cultured on the TNA samples. The apoptosis rate of Saos-2 cells on undoped TNA, 0.5 wt%, 5 wt%, and 22 wt% nano Se-doped TNA are 9.81%, 26.9%, 52.3%, and 16.19%, respectively (Figure 4C). Obviously, the apoptosis rate of 0.5 wt% and 5 wt% nano Se-doped TNA is significantly higher than that of undoped TNA and 22 wt% nano Se-doped TNA. It is concluded that the inhibitory effect of nano Se-doped TNA on osteosarcoma does not follow a dose-dependent behavior.

From the above results of the biological experiments, we suggest that the mechanism of nano Se inhibiting tumor cell proliferation is mainly the induction of cell apoptosis. It can be seen that the proliferation of Saos-2 cells on the surface of the nano Se-doped TiO_2_ nanotube arrays is significantly inhibited compared to that of the undoped TiO_2_ nanotube arrays. In this study, 0.5 wt% and 5 wt% nano Se-doped TiO_2_ nanotube arrays exhibit strong inhibition towards Saos-2 cell activity, especially for nano Se content of 5 wt%. The inhibitory effect of 22 wt% nano Se-doped TNA on osteosarcoma cells is less than that of lower concentration nano Se doped TNA. We believe that this inhibitory effect depends heavily on the content of the deposited nano Se and should be strongly related to the surface morphology and diameter of nano Se. We assume the selenium ions in the electrolyte are reduced to red elemental nano selenium, and the unique morphology of TNA guides the deposition of red elemental nano selenium. When the nano Se-doped concentration is 0.5 wt% and 5 wt%, the deposited nano Se is distributed on the surface of the nanotubes as nanoflakes with a diameter of about 100 nm, and these nanoflakes are not connected with each other, and EDS verifies that these nanoflakes are Se. Subsequently, Saos-2 cells ingested the nanoflakes into the cells to exert anti-tumor effects through endocytosis (Dai et al., 2021); when the doped nano Se concentration is 22 wt%, nano Se agglomerates into particles with a diameter of about 400 nm, covering the whole surface of the nanotube array (Figure 1B), due to the large size of the nanoparticles, tumor cells can only absorb trace amounts of nano-selenium on the surface of the nanotubes, resulting in relatively fewer apoptotic Saos-2 cells than the other two groups (Zhang et al., 2009). Therefore, we believe that nanoflake morphology of nano Se exhibit better anti-tumor effects than agglomerated Se particles. In addition, the effects of selenium on tumor cells varies within different types and numbers of tumor cells (Dennert and Horneber, 2006).

### 3.5 Identification of the apoptosis signaling pathway activated by nano Se-doped TNA

Previous studies have demonstrated that the antitumor effects of selenium compounds are mainly due to ROS produced by intracellular metabolism (Menon et al., 2018). Hence, we hypothesize that the reason why nano Se-doped TiO_2_ nanotube arrays induce apoptosis of osteosarcoma Saos-2 cells may be the production of ROS and the subsequent activation of endogenous and exogenous caspase-dependent pathways.

**FIGURE 5:**
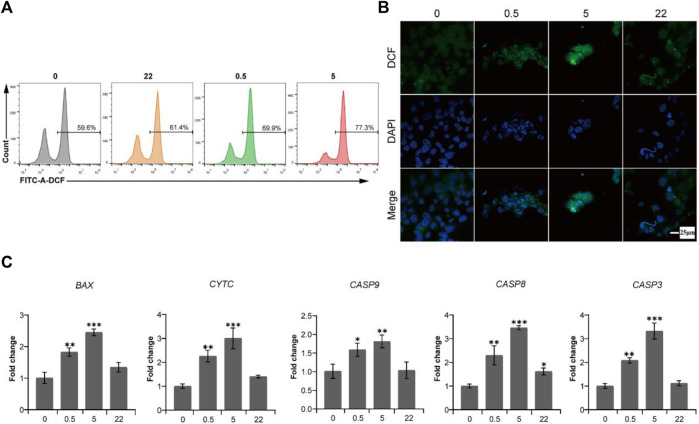
The production of ROS induced by deposited nano Se on TNA activates endogenous and exogenous caspase-dependent pathways to cause the apoptosis of osteosarcoma Saos-2 cells. **(A)** Flow cytometry data of ROS detection with the fluorescent probe DCFH-DA after Saos-2 cells culturing on the surface of TNA samples for 24 h. **(B)** Confocal laser scanning fluorescence microscopy images of ROS detection with the fluorescent probe DCFH-DA after Saos-2 cells culturing on the surface of TNA samples for 24 h. Scale bar = 25 μm. **(C)** mRNA expression of pro-apoptosis genes (*BAX*, *CYTC*, *CASP9*, *CASP8*, *CASP3*) of Saos-2 cells cultured on various samples. 0, 0.5, 5, 22 represent undoped TNA, 0.5 wt% nano Se-doped TNA, 5 wt% nano Se-doped TNA, 22 wt% nano Se-doped TNA, respectively. *n* = 3 independent samples, *p < 0.05, ** *p* < 0.01, *** *p* < 0.001, by one-way ANOVA.

To verify this hypothesis, we first measured the fluorescence intensity of 2′,7′-dichlorofluorescein (DCF) to reflect the intracellular ROS level after Saos-2 cells culturing on the surface of all TNA groups for 24 h. As shown in Figure 5A and Figure 5B, the ROS levels of the 0.5 wt% nano Se-doped TNA and 5wt% nano Se-doped TNA groups are higher than those of the undoped TNA and 22 wt% nano Se-doped TNA groups, and the ROS levels of the 5 wt% nano Se-doped TNA group are significantly higher than those of the other three groups.

It is well known that caspase-3, as a cell apoptotic executor, can be activated by both caspase-8 and caspase-9. Caspase-8 is the apoptosis promoter of the exogenous pathway (death receptor pathway), whereas caspase-9 is the apoptosis promoter of the endogenous pathway (mitochondrial pathway) (Mata et al., 2016). To demonstrate the potential caspase-dependent apoptosis mechanism, RT-PCR is used to detect the expression levels of pro-apoptotic genes of endogenous and exogenous caspase-dependent pathways after Saos-2 cells culturing on all TNA groups for 24 h. As shown in Figure 5C, the expression of promoting apoptosis genes (*BAX*, *CYTC*, *CASP9*, *CASP8*, *CASP3*) of 0.5 wt% nano Se-doped TNA group and 5%wt nano Se-doped TNA group is remarkably upregulated, compared with undoped TNA and 22 wt% nano Se-doped TNA group. In this study, we reveal a potential intrinsic mechanism, namely caspases-dependent apoptosis, regarding the inhibitory effect of nano selenium-doped TNA on tumor.

**FIGURE 6:**
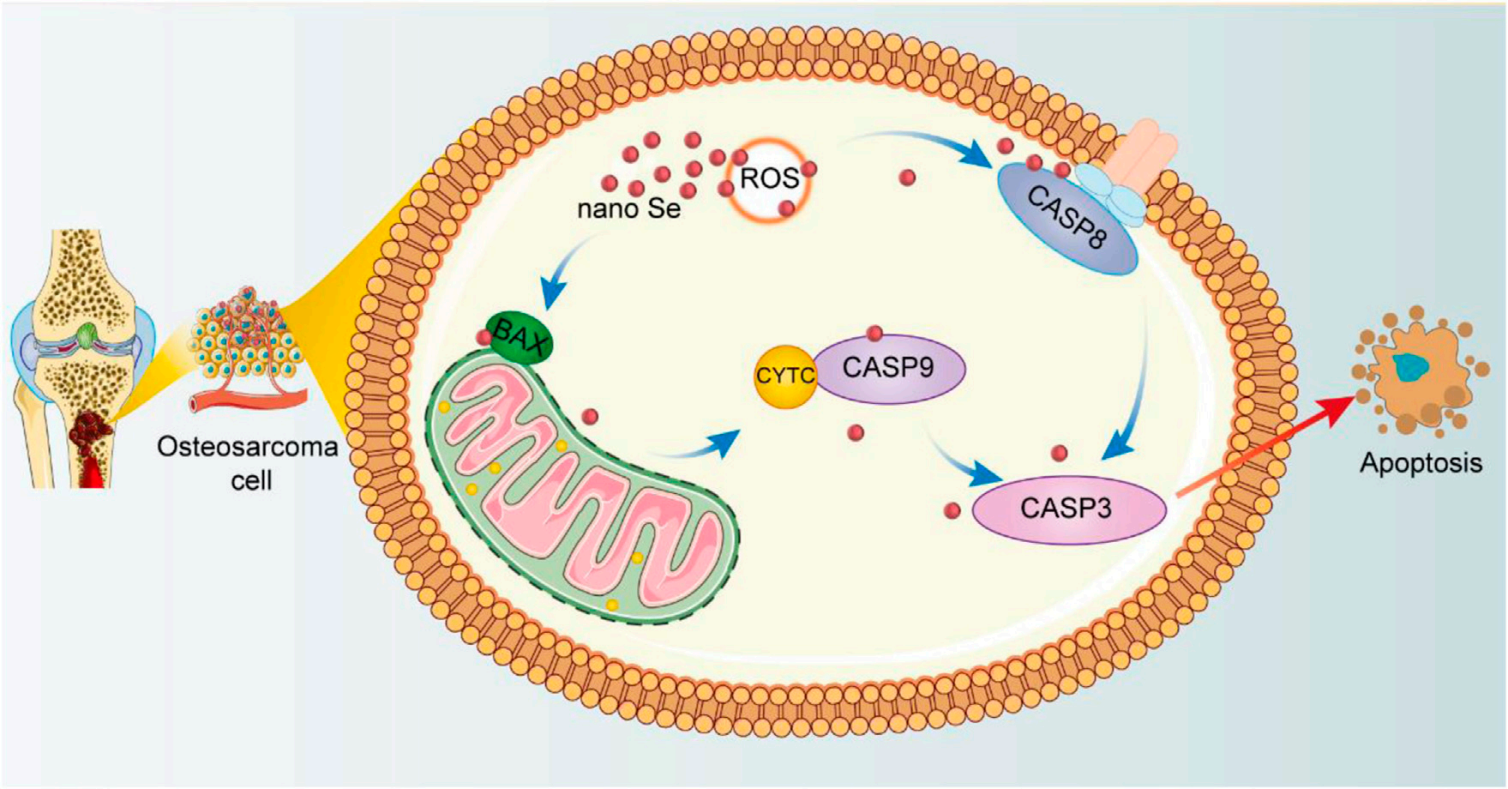
Schematic illustration of the anti-cancer effect of nano Se-doped TNA.

In summary, we can provide a clearer picture of the mechanism of nano Se-doped TNA inducing apoptosis of Saos-2 cells (Figure 6). The nano Se-doped TNA enters osteosarcoma cells via the endocytic pathway (Zhang et al., 2009; Wang et al., 2016; Dai et al., 2021). Subsequently, nano Se-doped TNA inhibits osteosarcoma cell proliferation by inducing the production of ROS, which subsequently activates two caspase-dependent apoptotic pathways, endogenous and exogenous, leading to apoptosis of osteosarcoma cells. On the one hand, ROS production activates caspase-8 and triggers the death receptor-mediated exogenous apoptotic pathway, while on the other hand, activation of the Bcl-2 family pro-apoptotic member (*BAX*) causes mitochondrial outer membrane permeabilization, resulting in the release of cytochrome C and effective activation of the endogenous apoptotic pathway, and ultimately resulting in the apoptosis of osteosarcoma cells.

## 4 Conclusion

In this study, nano Selenium is successfully deposited on the surface of anodized TiO_2_ nanotube arrays by electrochemical deposition. The low concentration of Se are deposited on the surface of TiO_2_ nanotube arrays as nanoflake morphology, and with the increase of Se concentration on the surface of TNA, Se elements gradually agglomerated into a film layer covering the surface of TNA. In addition, the concentration of doped Se on the surface of TNA can be adjusted by tuning parameters, such as electrolyte concentration and deposition time, and the concentration of doped Se increases with the increase of electrolyte composition concentration and deposition time. Cell evaluation *in vitro* show that nano Se-doped TNA exhibit significant anti-tumor effect and remarkably induce apoptosis in human osteosarcoma cells. This anti-osteosarcoma ability of Se-doped TNA is strongly related to the doping concentration of Se. 0.5 wt% and 5 wt% nano Se-doped TNA groups exhibit stronger anti-tumor effect compared with that of undoped TNA group and 22 wt% nano Se-doped TNA group, while the 5wt% nano Se-doped TNA group shows the strongest inhibition among all TNA groups. Finally, we reveal systematically the mechanism of tumor cell apoptosis induced by nano Se-doped TNA. Specifically, nano Se induces ROS production in osteosarcoma cells and subsequently activates endogenous and exogenous caspase-dependent apoptotic pathways.

## Data Availability

The raw data supporting the conclusion of this article will be made available by the authors, without undue reservation.
