# Supplementary material for: Nano selenium-doped TiO2 nanotube arrays on orthopedic implants for suppressing osteosarcoma growth
Source: Front Bioeng Biotechnol. 2023 Sep 5;11:1252816. doi: 10.3389/fbioe.2023.1252816 (PMC10508061; doi:10.3389/fbioe.2023.1252816)
Supplement: Supplementary file 1 [file DataSheet1.PDF]

Supplementary Material

Nano selenium-doped TiO_2 nanotube arrays on orthopedic implants for suppressing osteosarcoma growth

Xiaodong Hu[a, 1], Chunhai Ke[a, 1], Jiaqi Zhong[a], Yujiong Chen[a], Jieyang Dong[a], Mingming Hao[c], Qi Chen[b], Jiahua Ni[b*], Zhaoxiang Peng[a*]

[a] The Affiliated Lihuili Hospital, Ningbo University, Ningbo 315040, China

[b] Ningbo Regen Biotech, Co., Ltd., 168 East Hexiao Road, Ningbo, Zhejiang, 315100, China

[c] Ningbo Institute of Innovation for Combined Medicine and Engineering (NIIME), Ningbo Medical Centre Lihuili Hospital, Ningbo University, Ningbo 315100, China

***** Correspondence:

Zhaoxiang Peng

The Affiliated Lihuili Hospital, Ningbo University, 57 Xingning Road, Ningbo 315040, People's Republic of China

Tel +86 574 8701 8511

Fax +86 574 8701 8701

Email: pzxao@hotmail.com

Jiahua Ni

Ningbo Regen Biotech, Co., Ltd., 168 East Hexiao Road, Ningbo 315100, China

Tel +86 574 8723 6677

Fax +86 574 8723 6677

Email: jhni2022@163.com

[1] Co-first authors: These authors contributed equally to this work.

Supplementary Figures

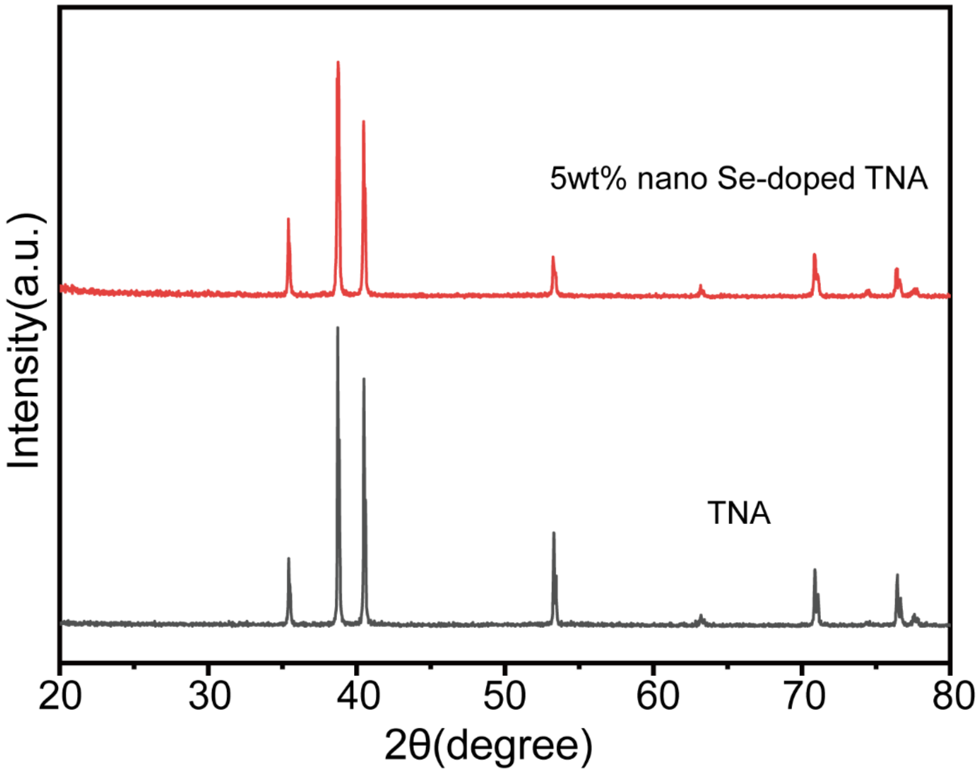

Figure S1: XRD spectra.
